# Serum Levels of Irisin Are Positively Associated with Improved Cardiac Function in Patients with Heart Failure with Reduced Ejection Fraction

**DOI:** 10.3390/biomedicines13040866

**Published:** 2025-04-03

**Authors:** Alexander E. Berezin, Tetiana A. Berezina, Evgen V. Novikov, Oleksandr O. Berezin

**Affiliations:** 1 Division of Cardiology, Department of Internal Medicine II, Paracelsus Medical University, 5020 Salzburg, Austria; 2 VitaCenter, Department of Internal Medicine and Nephrology, 69000 Zaporozhye, Ukraine; talexberezina@gmail.com; 3 Department of Functional Diagnostics, Shupyk National Healthcare University of Ukraine, 04136 Kyiv, Ukraine; doctornovikov@ukr.net; 4 Luzerner Psychiatrie AG, 4915 St. Urban, Switzerland; lunik.mender@gmail.com

**Keywords:** heart failure, improved ejection fraction, cardiac function, irisin, natriuretic peptides, biomarkers

## Abstract

**Background**: The purpose of the study is to investigate a possible predictive value of irisin for improved left ventricular (LV) ejection fraction (EF) in discharged patients with known heart failure with reduced ejection fraction (HFrEF). **Methods**: We included in the study 313 patients who were discharged with HFrEF (at admission, LVEF ≤ 40%) and monitored for 3 months. HF with improved LVEF (HFimpEF) was characterized as a >40% increase in LVEF on transthoracic B-mode echocardiography within 3 months of follow-up. Circulating biomarkers including NT-proBNP and irisin were detected at baseline and after 3 months of observation. By the third month, 117 (37.4%) patients had HFimpEF, whereas 196 individuals were categorized as having persistent HFrEF. **Results**: We found that HFimpEF was related to lower LV end-diastolic dimensions and concentrations of NT-proBNP and higher left atrial volume index (LAVI) and irisin concentrations than those with persistent HFrEF. The most balanced cut-offs of irisin and NT-proBNP concentrations (improved LVEF versus non-improved LVEF) were 10.8 ng/mL and 1540 pmol/L, respectively. Multivariate regression analysis showed that atrial fibrillation (odds ratio [OR] = 0.95; *p* = 0.010), LAVI < 39 mL/m^2^ (OR = 1.23; *p* = 0.001), irisin levels ≥ 10.8 ng/mL (OR = 1.73; *p* = 0.001), and NT-proBNP < 1540 pmol/mL (OR = 1.47; *p* = 0.001) independently predicted HFimpEF. The discriminative ability of irisin ≥ 10.8 ng/mL was better than NT-proBNP < 1540 pmol/mL; the predictive ability of irisin alone was not improved by the combined model (irisin added to NT-proBNP). **Conclusions**: serum irisin ≥ 10.8 ng/mL predicted HFimpEF independently of natriuretic peptide in HFrEF patients.

## 1. Introduction

Despite the introduction of new diagnostic, preventive, and treatment options into routine clinical practice, heart failure (HF) remains a condition with unacceptably high mortality rates ranging from 0.2% to 17.7% in the general population [1,2]. Several population-representative studies have shown that one-year mortality from heart failure varied from 4% to 45%, with an average of 33% overall and 24% depending on the presentation of HF phenotypes, concomitant comorbidities across all adult age groups, and the categorization of left ventricular (LV) ejection fraction (EF) [3,4]. Interestingly, cardiovascular (CV) and HF readmission rates were markedly higher in patients with HF with reduced ejection fraction (HFrEF) and mildly reduced ejection fraction (HFmrEF) compared with those with HF with preserved ejection fraction (HFpEF), whereas the composite endpoint (all-cause mortality, cardiovascular death, and re-hospitalization) was similar in all subgroups [5,6,7].

With personalized guideline-directed medical therapy (GDMT), a significant proportion of patients with HFrEF show improvement in LVEF (known as HF with improved EF [HFimpEF]) [8]. Improved short and long-term clinical outcomes, including mortality and hospital readmissions, are associated with the recovery of systolic function in patients with HFrEF [9,10]. Despite the presence of several factors as positive predictors of HFimpHF, i.e., female gender, age < 55 years, de novo HF associated with hypertension and atrial fibrillation, sodium–glucose cotransporter-2 (SGLT2) inhibitors, and beta-blockers, and those as negative predictors, such as NYHA class III/IV, anemia, coronary artery disease (CAD), and type 2 diabetes mellitus (T2DM), the role of conventionally used circulating biomarkers in the management of HFimpHF is still not clearly investigated [10,11]. Moreover, the clinical benefit of contemporary management in terms of HFimpHF was present regardless of baseline N-terminal brain natriuretic pro-peptide (NT-proBNP) concentration, while higher NT-proBNP levels were correlated with a greater risk of a poor outcome [12]. However, post-discharge monitoring of NT-proBNP levels showed no clear benefit in predicting re-admission rate or non-HF adverse clinical events but was markedly associated with a lower risk of CV death [13]. Another aspect is the determination of non-HF mortality in HFimpHF after discharge from the hospital because non-cardiac causes accounted for more than 60% of deaths, which are not completely predicted by biomarkers of mechanical stress (NT-proBNP), myocardial damage (high-sensitivity troponin T/I), inflammation (galectin-3, interleukin-6, high-sensitivity C-reactive protein, growth differential factor-15), and fibrosis (soluble suppression of tumorigenicity 2 [sST2]) [14,15,16,17,18]. In addition, the improvement in the systolic function of the LV is associated with a reduction in the levels of serum natriuretic peptides [19]. A significant proportion of these patients frequently determined as having euvolemic status may have low/near-normal NT-proBNP concentrations (<300 pg/mL), which severely limits the ability to discriminate clinical outcomes in HFimpEF, so a large number of these patients may have an underestimated risk of adverse outcomes [20]. In this context, the discovery of new approaches based on circulating biomarkers for predicting HFimpEF could be considered promising.

In previous years, there has been considerable evidence that the intensity of systemic inflammation may be associated with severity and poor prognosis in all HF phenotypes [21,22]. In this context, CV risk factors (age, smoking), metabolic comorbidities (diabetes mellitus, dyslipidemia, obesity, thyroid dysfunction), cardiac cachexia, rheumatic and respiratory diseases, acute and chronic kidney disease (CKD), and altered microbiota contribute to HF occurrence through modulating inadequate immune response, mediating synthesis, and releasing inflammatory mediators, which consequently induce adipose tissue and skeletal muscle dysfunctions and intervene in vascular and cardiac remodeling [23].

**Figure 1:**
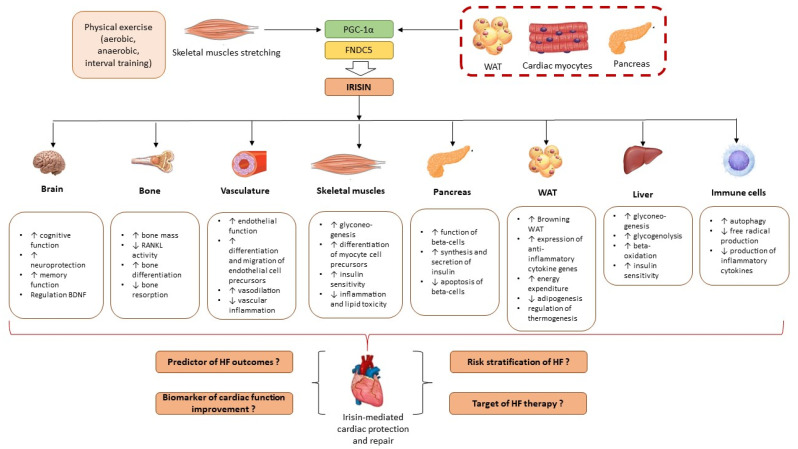
Rationale for investigating irisin in HF patients based on its biological effects. Abbreviations: BDNF, brain-derived neurotrophic factor; HF, heart failure; FNDC5, fibronectin type III domain-containing 5 protein; PGC-1α, peroxisome proliferator-activated receptor γ coactivator-1α; RANKL, receptor activator of nuclear factor -κB ligand; WAT, white adipose tissue; ↑, increase; ↓, decrease.

Irisin is the membrane-associated component of the fibronectin type III domain-containing 5 protein (FNDC5), which is activated by peroxisome proliferator-activated receptor γ (PPARγ) coactivator-1α (PGC-1α) after stretching in skeletal myocytes [24]. Several forms of physical exercise, i.e., aerobic, anaerobic, and interval training, have been identified as primary causes of increased irisin synthesis and secretion in circulation. In physiological conditions, irisin acts as a regulator of tyrosine residues insulin receptor phosphorylation, which increases the activity of the phosphatidylinositol 3-kinases pathway, thereby attenuating insulin sensitivity, inflammatory response, and cognitive function [25]. FNDC5/irisin stimulates the transcriptional potency RUNX1/2 factors through a focal adhesion kinase-dependent pathway in brain and peripheral tissues including bone and subcutaneous white adipose tissue (WAT) and consequently plays a pivotal role in thermogenesis, energy metabolism, and browning WAT [26,27]. In pathophysiological conditions, irisin is not only produced by skeletal myocytes but also adipocytes, cardiac myocytes, hepatocytes, and astrocytes [28,29]. Irisin prevents ischemia-reperfusion injury and cell necrosis/apoptosis/pyroptosis and protects against autophagy and mitochondrial dysfunction as well as reducing extracellular matrix accumulation, fibrosis, oxidative stress, and inflammation via the AKT/mTOR signaling, ERK1/2, and Sirtuin-1-p53-SLC7A11/GPX4 pathways [30,31,32]. The biological activities of irisin in physiological and pathophysiological conditions are reported in Figure 1.

A decrease in circulating irisin levels was not only associated with cardio- and cerebrovascular conditions and diseases (myocardial infarction, HF, hypertension, atherosclerosis, vascular dementia, stroke), osteoporosis, T2DM, obesity, and CKD but also demonstrated its discriminatory ability for the prognosis of their natural evolution [33,34,35,36,37,38]. Finally, the predictive potency of irisin concentrations was better than NT-proBNP in HF in patients with metabolic comorbidities, such as obesity and diabetes mellitus [39]. However, the possible role of irisin in predicting functional cardiac recovery in HFrEF patients remains not completely clear. The purpose of the study is to investigate the discriminatory ability of irisin for improved LVEF in discharged individuals with HFrEF.

## 2. Materials and Methods

### 2.1. Patient Population and Structure of the Study

**Figure 2:**
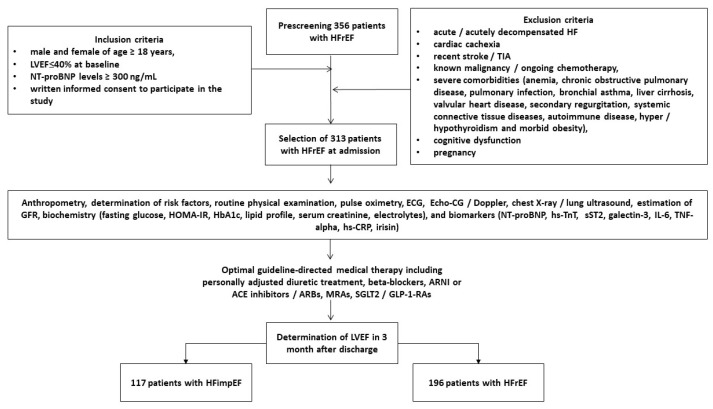
Flow chart of the study design. Abbreviations: ACE, angiotensin-converting enzyme; ARBs, angiotensin-II receptor blockers; ARNI, angiotensin receptor neprilysin inhibitors; Echo-CG, echocardiography; ECG, electrocardiography; GLP-1-RAs, glucagon-like peptide-1 receptor agonists; IL, interleukin; LVEF, left ventricular ejection fraction; hs-CRP, high-sensitivity C-reactive protein; HF, heart failure; HFrEF, heart failure with reduced ejection fraction; HFimpEF, heart failure with improved ejection fraction; MRAs, mineralocorticoid receptor antagonists; NT-proBNP, N-terminal brain natriuretic pro-peptide; TNF-alpha, tumor necrosis factor-alpha; sST2, soluble suppression of tumorigenicity-2; SGLT2, sodium–glucose co-transporter-2; TIA, transient ischemic attack.

We selected 356 in-patients with HFrEF who had hemodynamically stable status with NT-proBNP levels > 300 pmol/mL. Among them, 313 individuals met the inclusion criteria and were consecutively enrolled from October 2020 to November 2024. The subjects were then evaluated during a subsequent inpatient stay at the private hospital “Vita Center” (Zaporozhye, Ukraine), and the inclusion and exclusion criteria, in addition to the study procedures and the determination of clinical outcomes, are outlined in Figure 2. Patients with acute or acutely decompensated HF and HF de novo during hospitalization were not included in the study. However, the main reason for hospitalization was the initiation of optimal GDMT.

All patients were administered optimal GDMT, including personalized doses of diuretics, renin–angiotensin–aldosterone system inhibitors, beta-blockers, sodium–glucose cotransporter 2 (SGLT2) inhibitors, and glucagon-like peptide-1 (GLP-1) receptor agonists. At 3 months post-discharge, patients meeting the criteria for HFimpHF were enrolled in the first cohort (*n* = 117), whereas those without improvement in LVEF were categorized as having persistent HFrEF and enrolled in the second cohort (*n* = 196).

### 2.2. The Evaluation of Participants’ Demographics, Anthropometry Parameters, and Concomitant Diseases/Conditions

The study collected detailed information about the participants’ demographics (age, gender) and anthropometry parameters including weight and body mass index (BMI). The evaluation involved an assessment of conventional CV risk factors and concomitant diseases/conditions, such as hypertension, smoking, dyslipidemia, obesity, atrial fibrillation, CAD, T2DM, and CKD.

### 2.3. Determination of HFimpEF

**Figure 3:**
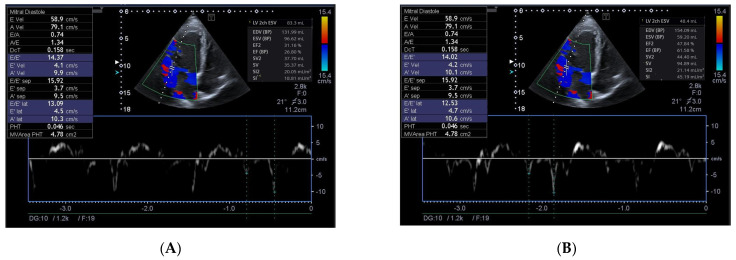
An example of the determination of HFimpEF in patients with a 3-month interval between investigations. (**A**) Echocardiographic parameters at baseline. (**B**) Echocardiographic parameters in 3 months.

HFimpEF was defined as an improvement in LVEF from ≤40% at baseline to >40% (with an increase of ≥10%) within 3 months of discharge from the hospital [11]. Figure 3 reports echograms received from the patients with LVEF < 40% at baseline (Figure 3A) and with LVEF > 40% in 3 months (Figure 3B).

### 2.4. Echocardiography Examination

Standard B-mode transthoracic echocardiography in apical 2- and 4-chamber views using a GE Healthcare Vivid E95 scanner (General Electric Company, Strandpromenaden 45, 3183 Horten, Norway) was performed by highly qualified assessors in accordance with the 2018 Guideline of the American Society of Echocardiography [40]. This encompassed cardiac dimensions, LV end-diastolic (LVEDV) and end-systolic (LVESV) volumes, left atrial volume index (LAVI), tricuspid annular plane systolic excursion (TAPSE), and LVEF by Simpson’s method. Doppler examination was performed to determine the presence of mitral and tricuspid regurgitation and to measure early diastolic mitral blood filling (E) and medial and lateral e` velocities. The averaged medial e` and lateral e` velocities were estimated to obtain E/e`. LV hypertrophy was defined as an LV mass index (LVMI) of ≥95 g/m^2^ or ≥115 g/m^2^ in women and in men, respectively [40].

### 2.5. Glomerular Filtration Rate and Insulin Resistance Determination

The CKD-EPI formula was applied to estimate the glomerular filtration rate (eGFR) [41]. The Homeostatic Assessment Model of Insulin Resistance (HOMA-IR) was employed to evaluate insulin resistance [42].

### 2.6. Circulating Biomarker Evaluation

Peripheral blood samples were obtained via venipuncture and centrifuged at 3000 rpm for a period of 10 min with further storing of harvested serum at a temperature of −70 °C until evaluation.

The routine hematological and biochemical parameters were determined using a Roche P800 analyzer (Basel, Grenzacherstrasse 124, Switzerland) in the Private Medical Center “Vita Centre” (Zaporozhye, Ukraine).

Circulating biomarkers (tumor necrosis factor [TNF]-alpha, high-sensitive C-reactive protein [hs-CRP], high-sensitive troponin T [hs-TnT], NT-proBNP, interleukin [IL]-6, galectin-3, soluble suppression of tumorigenicity-2 [sST2], irisin) were measured in serum samples using ELISA kits (Elabscience, 14780 Memorial Drive, Suite 105, Houston, TX, USA) according to the manufacturer’s instructions. Each blood sample was analyzed on two separate occasions, and the mean of these measurements was used for the final evaluation. The coefficient of variability for each marker was found to be less than 10% for both the intra- and inter-assay comparisons.

### 2.7. Statistics

Statistical analysis of the data was conducted utilizing SPSS Statistics 29 (IBM, 1 Orchard Road, Armonk, NY, USA) and Prism v.10 (GraphPad, 2365 Northside Dr., Ste. 560, San Diego, CA, USA) software. Variables are reported as mean (M) and standard deviation (SD), median (Me), and interquartile range (IQR) or absolute numbers (*n*) and percentages (%) as appropriate. The Anderson–Darling test was employed to verify the data distribution. Continuous variables were compared using the paired t-test or the Mann––Whitney test, where appropriate, while categorical variables were compared using Fisher’s exact test. Univariate logistic regression and backward stepwise multivariate logistic regression were used to identify potential predictors of HFimpEF, with odds ratios (ORs) and 95% confidence intervals (CIs) calculated for each. Factors with a *p* less than 0.05 in the univariate log regression analysis were included in the multivariate log regression model. For each predictor, the area under the curve (AUC), its confidence interval (CI), sensitivity (Se), and specificity (Sp) were calculated using receiver operating curve (ROC) analysis. The Youden test was used to estimate the cut-off points for serum levels of TNF-alpha, galectin-3, sST2, hs-CRP, irisin, and NT-proBNP. The integrated discrimination indices (IDI) and net reclassification improvement (NRI) were also estimated to assess the discriminatory ability of the predictive models. Comparisons with a *p*-value of less than 0.05 were considered statistically significant.

## 3. Results

### 3.1. Clinical Characteristics, Echocardiographic Parameters and Laboratory Findings

The study included 117 patients with HFimpEF and 196 individuals with persistent HFrEF. The characteristics of the enrolled individuals are presented in Table 1. The mean age of the patients in this study was 69 years, and 58.9% were male. The patients had comorbidity profiles that included smoking (43.1%), obesity (24.0%), dyslipidemia (74.8%), hypertension (56.2%), CAD (51.8%), dilated cardiomyopathy (18.2%), atrial fibrillation (29.7%), CKD 1–3 grades (21.7%), and T2DM (32.6%). Percutaneous coronary intervention was provided to about 31.0% of the patients from the entire group. Along with it, NYHA HF classes II, III, and IV were detected in 23.0%, 58.9%, and 18.1%, respectively. All patients had stable hemodynamics and increased LV diastolic and systolic volumes, as well as an LVEF of less than 40% at baseline. The patients had dyslipidemia, mild increases in HOMA-IR, circulating creatinine, inflammatory biomarkers (hs-CRP, TNF-alpha, IL-6), and indicators of fibrosis (galectin-3 and sST2). The mean serum levels of NT-proBNP and irisin were 1810 pmol/mL and 5.75 ng/mL, respectively. Concomitant medications included RAAS inhibitors, SGLT-2 inhibitors, mineralocorticoid receptor antagonists (MRA), beta-blockers, ivabradine (for those with sinus rhythm and uncontrolled heart rate on beta-blocker therapy), diuretics, statins, and anticoagulants/antiplatelet agents. Patients with concomitant T2DM were treated with a personalized diet, metformin, and/or GLP-1 receptor agonists to reach full control of glycemia.

No significant differences were found between groups in age, gender, BMI, waist circumference, smoking, obesity, dyslipidemia, hypertension, dilated cardiomyopathy, LV hypertrophy, and T2DM. The proportions of current stable CAD, atrial fibrillation, CKD grade 1–3, and PCI history were significantly higher in patients with HFrEF compared to HFimpEF. There were no differences in the presentation of NYHA HF classes between the two patient cohorts.

Patients with HFimpEF had significantly lower LVEDV, LVESV, and LAVI than those with HFrEF. TAPSE, E/e`, and LVMI did not differ between the two cohorts. Along with it, eGFR, creatinine, electrolytes, lipid profile, HOMA-IR, fasting glucose, HbA1c, hemoglobin, hematocrit, serum uric acid, hs-CRP, TNF-alpha, IL-6, cTnT, sST2, and galectin-3 were not sufficiently different between cohorts. In contrast, NT-proBNP levels were higher and irisin concentrations were lower in HFimpEF compared with HFrEF. With the exception of anticoagulants, there were no differences in concomitant medications between the cohorts.

### 3.2. The Optimal Cut-Offs for Possible Predictors of HFimpEF

**Figure 4:**
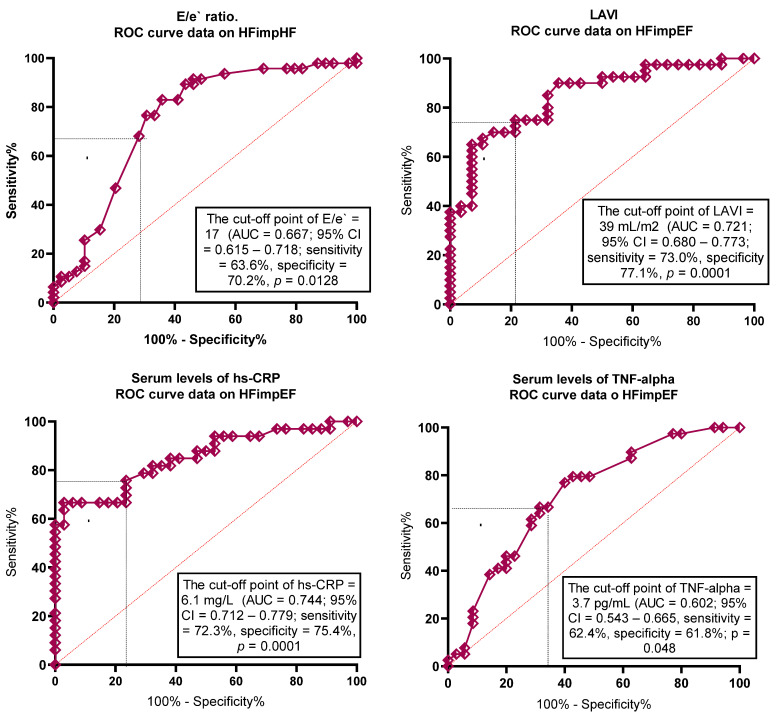

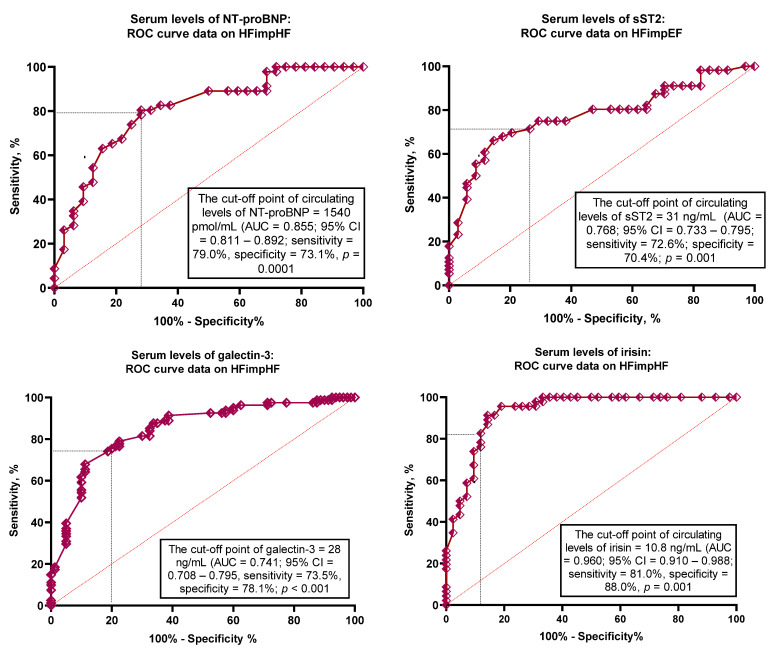
Receiver operating characteristic curves for predictive factors of HFimpHF. Abbreviations: AUC, area under curve; CI, confidence interval; hs-CRP, high-sensitivity C-reactive protein; E/e`, early diastolic blood filling to longitudinal strain ratio; LAVI, left atrial volume index; Se, sensitivity; Sp, specificity; NT-proBNP, N-terminal brain natriuretic pro-peptide; sST2, soluble suppression of tumorigenicity-2; TNF-alpha, tumor necrosis factor-alpha.

To determine the optimal cut-offs for potential predictors of HFimpEF, ROC curve analysis was performed (Table 2). The following factors for further regression analysis have been identified: LAVI < 39 mL/m^2^, E/e` < 17; hs-CRP < 6.1 mg/L; TNF-alpha < 3.7 ng/mL; NT-proBNP < 1540 pmol/mL; sST2 < 31 ng/mL, galectin-3 < 28 ng/mL; irisin >10.8 ng/mL) (Table 2). The ROC curves are reported in Figure 4. The AUCs of the biomarkers show a high degree of reliability of the models.

### 3.3. Predictive Factors for HFimpEF: Univariate Logistic Regression and Backward Stepwise Multivariate Logistic Regression Models

Univariate logistic regression showed that atrial fibrillation, CKD stages 1–3, LAVI < 39 mL/m^2^, E/e` < 17, hs-CRP < 6.1 mg/L, TNF-alpha < 3.7 ng/mL, NT-proBNP < 1540 pmol/mL, sST2 < 31 ng/mL, galectin-3 < 28 ng/mL, and irisin > 10.8 ng/mL were associated with improved LVEF in individuals with HF (Table 3). Backward stepwise multivariate logistic regression revealed that the presence of AF, LAVI < 39 mL/m^2^, NT-proBNP < 1540 pmol/mL, and irisin ≥ 10.8 ng/mL were independent predictors for HFimpEF.

### 3.4. Prediction Model Comparison

Model 1 (NT-proBNP < 1540 pmol/mL) and model 2 (a presence of AF) did not markedly differ from one another in the prediction of HFimpEF, whereas model 3 (LAVI < 39 mL/m^2^) was worse than the reference model (Table 4). Model 4 (irisin ≥ 10.8 ng/mL) was significantly better than model 1. Furthermore, the combined model 1 + 4 (NT-proBNP < 1540 pmol/mL + irisin ≥ 10.8 ng/mL) was superior to model 1 alone (NT-proBNP < 1540 pmol/mL) but did not sufficiently increase the discriminatory power of model 4 (irisin ≥ 10.8 ng/mL). Other combined models showed no better benefit for HFimpEF than model 1.

## 4. Discussion

This study has revealed that positive changes in hemodynamics among optimally treated patients with HFimpEF were associated with a decline in serum levels of NT-proBNP and an increase in circulating levels of irisin, whereas the concentrations of conventionally used biomarkers reflecting inflammation (hs-CRP, IL-6, TNF-alpha), fibrosis (galectin-3, sST2), and cardiac damage (hs-TnT) remained comparable between the groups of individuals with HFimpHF and persistent HFrEF. Moreover, serum levels of irisin ≥ 10.8 ng/mL added new predictive information to NT-proBNP for HFimpEF, but the combination of these two biomarkers did not demonstrate its discriminative benefit in comparison with irisin alone.

As HFimpEF relates to a 50% greater reduction in mortality risk and hospitalization compared with HFrEF, promoting improvement in LVEF and predicting a positive response to treatment are both sides of the same coin [43]. Indeed, the prognosis of almost a quarter of patients with HFrEF can be significantly improved in case of sustained improvement in myocardial contractility assessed as LVEF > 40% [43,44]. Notably, recent clinical studies have identified a number of promising positive (NYHA Class II HF, concomitant hypertension, beta-blocker use) and negative (alcohol consumption, dilated cardiomyopathy, serum uric acid) predictors of LVEF improvement [44,45]. However, these studies did not include patients receiving optimal therapy for HFrEF based on four major classes of prognosis-improving drugs. In our study, despite significant comorbidity, all patients received personalized optimal GDMT for HFrEF with any RAAS inhibitor, MRA, beta-blockers, and SGLT2 inhibitors. This resulted in 37.4% of patients in the HFrEF group having a positive response to HF therapy, including a reduction in LVEDV and LVESV, an increase in LVEF, and the control of circulating NT-proBNP (<2000 pmol/mL). These findings support previous evidence of clinical trials and current guideline recommendations for the use of GDMT in patients with HFimpEF [11,46,47].

Another aspect of the study was that atrial fibrillation and CKD were the most significant comorbidities for the prediction of HFimpEF, whereas in some studies and meta-analyses, not only the abovementioned conditions but also anemia, T2DM, CAD, dyslipidemia, cerebrovascular disease, and hypertension were found to be predictive factors for recovered LVEF [48,49]. However, the signature of comorbidities often depends on the age and gender of the patients. In our study, age was not identified as a negative predictive factor for HFimpEF, probably because related comorbidities also did not show a high association with the recovery of LV contractile function in the short term (about 3 months). It is conceivable that the profile of comorbid factors with plausible predictive values for HFimpEF may change as patient follow-up is extended to one year or even longer.

Nevertheless, the results open one of the most important aspects of the debate around whether NT-proBNP retains its prognostic value in patients with HFimpEF. It has previously been shown that low or near-normal concentrations of NT-proBNP are unlikely to accurately discriminate clinical outcomes in patients with HFpEF [50]. This should not necessarily mean that well-controlled levels of natriuretic peptides lose their importance in HF patients with the recovery of systolic function during guideline-based treatment. However, there is evidence that alternative biomarkers such as irisin can significantly increase the discriminatory potency of NT-proBNP [51]. Our data clearly show that irisin not only adds prognostic information to NT-proBNP but also outperforms NT-proBNP in its ability to predict HFimpEF.

Previous studies have shown that irisin has various influences on key points of the pathogenesis of HF, such as mitochondrial and endothelial dysfunction, vasoconstriction, oxidative stress, immune and inflammatory reactions, metabolic imbalance, skeletal muscle dystrophy, altered energy expenditure, and tissue reparation [29,52,53]. Therefore, low levels of irisin were found to be a negative factor with possible predictive ability for any HF phenotype [54,55,56]. However, it has remained unclear whether guideline-directed HF therapy is able to modulate serum irisin levels and whether HFimpEF is associated with the restoration of circulating irisin.

First, we have clearly shown that optimal therapy in HFrEF is related to a higher likelihood of improvement in LVEF and that the control of NT-proBNP in HFrEF may be associated with the restoration of irisin levels. It seems particularly valuable that the positive response in the form of improved LVEF with GDMT was not accompanied by significant changes in biomarkers reflecting myocardial injury (hs-TnT), inflammation (hs-CRP, IL-6, TNF-alpha), and fibrosis (sST2, galectin-3). Previously, the possibility of serially measuring these biomarkers to predict response to heart failure therapy and assess the risk of adverse complications has been widely debated. Nevertheless, as observed, no major differences were found between HFimpEF and persistent HFrEF groups at 3 months in our study. This may be partly due to the fact that the study included hemodynamically stable patients who did not require inotropic and mechanical support. It is for these groups of patients with HFrEF and acute or acutely decompensated HF that the predictive role of these biomarkers seems particularly relevant [57]. Although irisin and inflammatory biomarker concentrations are inversely correlated in untreated HFrEF patients or patients with acute HF, this relationship is apparently less pronounced in patients with HFimpEF. In any case, the mechanisms by which irisin may interfere with the recovery of myocardial systolic function need to be studied in detail in the future. Therefore, irisin levels should not only be considered as a plausible predictive biomarker but also as an additional target for the therapy of HF.

## 5. Clinical Implications

These findings are likely to support the integration of these advances into clinical practice in the future and highlight the need for ongoing clinical research to fully recognize their value to change HFrEF management. The findings of our study possibly open a new approach to predicting the recovery of LV systolic function. Although for most patients with different HF phenotypes, including HFrEF, a decrease in NT-proBNP levels indicates clinical outcomes and a reduced risk of re-hospitalization, it is not clear that a trend toward a decrease in circulating NT-proBNP concentration is associated with the recovery of LVEF and the occurrence of HFimpEF. Moreover, the high variability of serum NT-proBNP, especially pronounced in fluid retention, the progression of CKD, and/or T2DM, may require more frequent repeated measurements of its concentration, which inevitably negatively affects the cost of monitoring. In this context, the measurement of irisin not only improves the prognostic value of single measured NT-proBNP but also reduces the economic burden of predicting HFimpEF. Another important clinical advantage of the new approach is the independence of the predictive value of irisin from the presence of comorbid conditions such as CKD and T2DM. Thus, the creation of a new algorithm for predicting HFimpEF based on the measurement of irisin and NT-proBNP in patients with HFrEF is promising and requires further validation in a study with a larger sample size.

## 6. Study Limitations

The study includes several limitations. Although only patients receiving optimal GDMT were included in the study, it remains unknown whether the predictive effect of irisin in combination with NT-proBNP in HFimpEF is maintained when treatment is stopped or the doses of the drugs used are reduced. Another limitation is that we did not investigate the predictive value of irisin in individuals with normal or near-normal NT-proBNP concentrations. In addition, the lack of assessment of patients’ metabolic and nutritional status and the inclusion of HF patients with GDMT are other limitations of this study. However, we believe that these limitations do not influence the interpretation of the results.

## 7. Conclusions

**Figure 5:**
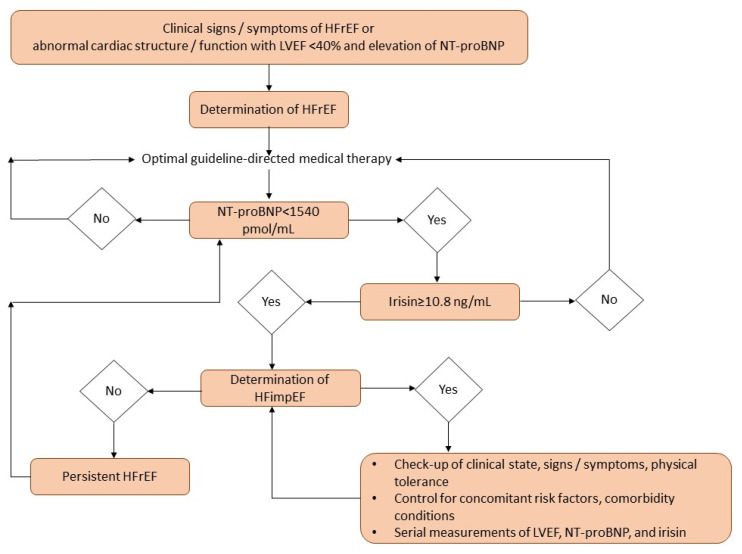
Possible predictive algorithm of HFimpEF with biomarker use. Abbreviation: HFrEF, heart failure with reduced ejection fraction; HFimpEF, heart failure with improved ejection fraction; LVEF, left ventricular ejection fraction; NT-proBNP, N-terminal brain natriuretic pro-peptide.

We have found that an improvement in LVEF in patients with HFrEF is closely associated with a decisive trend toward the restoration of serum irisin levels and a decrease in NT-proBNP. Serum irisin >10.8 ng/mL has been shown to be independent of NT-proBNP in predicting HFimpEF, while its use in conjunction with NT-proBNP did not sufficiently improve the discriminative value of irisin alone. These findings may see irisin emerge as a valuable biomarker for predicting improved LVEF in HFrEF patients, possibly modifying HF management. A possible algorithm for decision-making based on a comprehensive assessment of biomarker dynamics is presented in Figure 5.

It is likely that monitoring serum NT-proBNP and irisin could help in assessing the risk of HFimpEF destabilization and HFrEF persistence despite GDMT, although this assumption needs to be confirmed in further studies. However, the serial measurement of irisin may be important to personify GDMT in HFrEF or HFimpEF.

## Figures and Tables

**Table 1:** Baseline general characteristics of eligible patients.

Variables	Entire HF Patient Cohort (*n* = 313)	Patients with HFimpEF (*n* = 117)	Patients with HFrEF (*n* = 196)	*p* Value Between Cohorts
Demographics and anthropometry parameters				
Age, year	69 (61–78)	67 (60–75)	70 (62–81)	0.146
Male gender, *n* (%)	184 (58.9)	68 (58.1)	116 (59.2)	0.146
BMI, kg/m^2^	26.2 ± 4.26	25.3 ±3.88	26.9 ± 3.97	0.272
Waist circumference, cm	101 ± 7	99 ± 5	101 ± 8	0.690
Medical history				
Smoking history, *n* (%)	135 (43.1)	48 (41.0)	87 (44.4)	0.642
Abdominal obesity, *n* (%)	75 (24.0)	27 (23.1)	48 (24.5)	0.475
Dyslipidemia, *n* (%)	234 (74.8)	86 (73.5)	148 (75.5)	0.344
Hypertension, *n* (%)	176 (56.2)	66 (56.4)	110 (56.1)	0.871
Stable CAD, *n* (%)	162 (51.8)	57 (48.7)	105 (53.6)	0.046
Dilated cardiomyopathy, *n* (%)	57 (18.2)	20 (17.1)	37 (18.9)	0.242
Atrial fibrillation, *n* (%)	93 (29.7)	28 (23.9)	65 (33.2)	0.048
LVH, *n* (%)	217 (69.3)	81 (69.2)	136 (69.4)	0.844
CKD 1–3 grades, *n* (%)	68 (21.7)	22 (18.8)	46 (23.5)	0.044
T2DM, *n* (%)	102 (32.6)	38 (32.5)	64 (32.7)	0.526
PCI history, *n* (%)	97 (31.0)	42 (35.9)	55 (28.1)	0.048
NYHA functional class, *n* (%)				
II	72 (23.0)	29 (24.8)	43 (21.9)	0.142
III	184 (58.9)	69 (59.0)	115 (58.7)	0.416
IV	57 (18.1)	19 (16.2)	38 (19.4)	0.144
Hemodynamic and echocardiographic parameters				
Systolic BP, mm Hg	127 ± 8	129 ± 8	126 ± 9	0.395
Diastolic BP, mm Hg	68 ± 9	69 ± 7	68 ± 7	0.462
LVEDV, mL	176 (154–197)	178 (155–201)	173 (149–193)	0.274
LVESV, mL	103 (98–106)	99 (95–103)	110 (97–119)	0.022
LVEF, %	41 (34–51)	44 (40–47)	37 (33–39)	0.024
LVMI, g/m^2^	148 ± 22	147 ± 19	155 ± 20	0.226
LAVI, mL/m^2^	44 (35–54)	42 (36–49)	47 (40–53)	0.046
TAPSE, mm	20 (15–26)	19 (14–24)	22 (15–27)	0.611
E/e`, unit	17 ± 7	16 ± 4	17 ± 5	0.355
Laboratory findings				
eGFR, mL/min/1.73 m^2^	64 ± 19	65 ± 15	61 ± 13	0.331
K, mmol/L	4.1 (3.3–5.3)	4.3 (3.4–5.5)	4.0 (3.1–5.10)	0.124
Na, mmol/L	139 (128–146)	139 (125–149)	137 (127–145)	0.846
HOMA-IR, units	5.11 ± 2.33	5.05 ± 2.23	5.19± 2.25	0.658
Glucose, mmol/L	4.68 ± 0.57	4.59 ± 0.52	4.70 ± 0.51	0.681
HbA1c, %	5.10 ± 1.99	5.07 ± 1.65	5.11± 1.57	0.560
Hemoglobin, g/L	13.9 (12.6–15.1)	13.8 (12.5–14.7)	14.0 (12.6–15.3)	0.674
Hematocrit, %	38 (34–42)	38 (35–40)	39 (35–43)	0.644
Creatinine, µmol/L	104 ± 10	97 ± 11	106 ± 9	0.128
Serum uric acid, µmol/L	359 ± 85	352 ± 80	360 ± 88	0.672
TC, mmol/L	5.69 ± 0.60	5.61 ± 0.68	5.73 ± 0.66	0.654
HDL-C, mmol/L	1.02 ± 0.10	1.03 ± 0.09	1.02 ± 0.10	0.748
LDL-C, mmol/L	3.60± 0.20	3.50 ± 0.18	3.60± 0.20	0.786
Triglycerides, mmol/L	2.34 ± 0.37	2.30 ± 0.29	2.41 ± 0.27	0.650
hs-CRP, mg/L	5.98 (2.24–9.70)	5.52 (2.12–8.16)	6.11 (2.80–10.56)	0.860
TNF-alpha, pg/mL	3.68 (2.10–5.23)	3.45 (2.03–4.94)	3.81 (2.19–5.21)	0.547
IL-6, ng/mL	2.91 (0.76–4.95)	2.70 (0.67–4.82)	3.20 (0.88–5.61)	0.216
cTnT, ng/mL	0.036 (0.004–0.112)	0.021 (0.001–0.110)	0.048 (0.003–0.120)	0.690
NT-proBNP, pmol/mL	1810 (980–2560)	1330 (870–1580)	2310 (1130–3580)	0.044
sST2, ng/mL	29.40 (13.90–45.70)	27.63 (11.17–41.80)	31.90 (15.82–47.54)	0.844
Galectin-3, ng/mL	27.5 (11.6–53.4)	24.1 (9.8–41.5)	32.7 (10.1–60.3)	0.671
Irisin, ng/mL	5.75 (2.18–9.12)	8.23 (4.26–13.50)	4.37 (1.62–7.17)	0.001
Concomitant medications and devices				
ACE inhibitors, *n* (%)	122 (39.0)	43 (36.8)	79 (40.3)	0.519
ARBs, *n* (%)	39 (12.5)	20 (17.1)	19 (9.7)	0.050
ARNI, *n* (%)	152 (48.7)	54 (46.2)	98 (50.0)	0.538
Beta-blockers, *n* (%)	285 (91.1)	105 (89.7)	180 (91.8)	0.351
Ivabradine, *n* (%)	32(10.2)	10 (8.5)	22 (11.2)	0.271
CCBs, *n* (%)	35 (11.2)	11 (9.4)	24 (12.2)	0.164
MRA, *n* (%)	231 (73.8)	86 (73.5)	145 (74.0)	0.834
Diuretics, *n* (%)	298 (98.2)	112 (95.7)	186 (94.9)	0.877
Antiplatelet agents, *n* (%)	179 (57.2)	69 (59.0)	110 (56.1)	0.048
Anticoagulants, *n* (%)	93 (29.7)	28 (23.9)	65 (33.2)	0.048
Metformin, *n* (%)	97 (31.0)	36 (30.8)	61 (31.1)	0.713
SGLT2 inhibitors, *n* (%)	227 (72.5)	86 (73.5)	141 (71.9)	0.637
GLP-1-RAs, *n* (%)	34 (10.8)	13 (11.1)	21 (10.7)	0.511
Statins, *n* (%)	234 (74.8)	86 (73.5)	148 (75.5)	0.344
RCT, *n* (%)	22 (7.0)	9 (7.7)	13 (6.6)	0.766

Abbreviations: ACE, angiotensin-converting enzyme; ARBs, angiotensin-II receptor blockers; ARNI, angiotensin receptor neprilysin inhibitors; BMI, body mass index; CAD, coronary artery disease; CCBs, calcium channel blockers; CKD, chronic kidney disease; eGFR, estimated glomerular filtration rate; E/e`, early diastolic blood filling to longitudinal strain ratio; HDL-C, high-density lipoprotein cholesterol; hs-CRP, high-sensitivity C-reactive protein; GLP-1-RAs, glucagon-like peptide-1 receptor agonists; IL, interleukin; LDL-C, low-density lipoprotein cholesterol; LAVI, left atrial volume index; LVEDV, left ventricular end-diastolic volume; LVESV, left ventricular end-systolic volume; LVEF, left ventricular ejection fraction; LVMI, left ventricle myocardial mass index, LVH, left ventricular hypertrophy; MRA, mineralocorticoid receptor antagonist; RCT, resynchronized therapy; NT-proBNP, N-terminal brain natriuretic pro-peptide; TC, total cholesterol; TNF-alpha, tumor necrosis factor-alpha; sST2, soluble suppression of tumorigenicity-2; SGLT2, sodium-glucose co-transporter-2.

**Table 2:** Receiver operating characteristic curve analysis for possible predictive factors of HFimpHF.

Variables	AUC	95% CI	*p*-Value	Cut-Offs	Se, %	Sp, %
LAVI	0.721	0.680–0.773	0.001	39 mL/m^2^	73.9	77.1
E/e`	0.667	0.615–0.718	0.001	17	63.6	70.2
hs-CRP	0.744	0.712–0.779	0.001	6.1 mg/L	72.3	75.4
TNF-alpha	0.602	0.543–0.665	0.048	3.7 pg/mL	62.4	61.8
NT-proBNP	0.855	0.811–0.892	0.0001	1540 pmol/mL	79.0	73.1
sST2	0.768	0.733–0.795	0.001	31 ng/mL	72.6	70.4
Galectin-3	0.741	0.708–0.795	0.001	28 ng/mL	73.5	78.1
Irisin	0.960	0.910–0.988	0.0001	10.8 ng/mL	81.0	88.0

Abbreviations: AUC, area under curve; CI, confidence interval; hs-CRP, high-sensitivity C-reactive protein; E/e`, early diastolic blood filling to longitudinal strain ratio; LAVI, left atrial volume index; Se, sensitivity; Sp, specificity; NT-proBNP, N-terminal brain natriuretic pro-peptide; sST2, soluble suppression of tumorigenicity-2; TNF-alpha, tumor necrosis factor-alpha.

**Table 3:** Predictive factors for HFimpEF: univariate logistic regression and backward stepwise multivariate logistic regression.

Predictive Factors	Univariate Logistic Regression			Backward Stepwise Multivariate Logistic Regression		
	Odds Ratio	95% CI	*p* Value	Odds Ratio	95% CI	*p* Value
T2DM *	0.97	0.91–1.02	0.212	-		
PCI history *	0.95	0.89–1.13	0.437	-		
AF *	0.95	0.91–0.98	0.010	0.95	0.90–0.98	0.010
Stable CAD *	1.02	0.94–1.17	0.380	-		
CKD stages 1–3 *	0.93	0.87–0.99	0.047	0.95	0.89–1.01	0.177
Dilated CMP *	0.96	0.92–1.02	0.422	-		
LAVI < 39 mL/m^2^ vs. ≥39 mL/m^2^	1.32	1.15–1.56	0.001	1.23	1.11–1.39	0.001
E/e` < 17 vs. ≥17	1.18	1.04–1.35	0.012	1.10	1.00–1.27	0.052
hs-CRP < 6.1 mg/L vs. ≥6.1 mg/L	1.12	1.06–1.20	0.018	1.09	1.00–1.20	0.120
TNF-alpha < 3.7 bg/mL vs. ≥3.7 ng/mL	1.06	1.01–1.12	0.044	1.05	0.99–1.10	0.206
NT-proBNP < 1540 vs. ≥1540 pmol/mL	1.56	1.12–2.15	0.001	1.47	1.11–2.12	0.001
sST2 < 31 ng/mL vs. ≥31 ng/mL	1.24	1.02–1.65	0.048	1.20	1.00–1.68	0.086
Galectin-3 < 28 ng/mL vs. ≥28 ng/mL	1.17	1.01–1.43	0.050	1.12	1.00–1.27	0.064
Irisin ≥ 10.8 ng/mL vs. <10.8 ng/mL	1.75	1.22–4.32	0.001	1.73	1.16–4.18	0.001

Abbreviations: AF, atrial fibrillation; CAD, coronary artery disease; CI, confidence interval; hs-CRP, high-sensitivity C-reactive protein; CKD, chronic kidney disease; LAVI, left atrial volume index; NT-proBNP, N-terminal brain natriuretic pro-peptide; sST2, soluble suppression of tumorigenicity-2; TNF-alpha, tumor necrosis factor-alpha; T2DM, type 2 diabetes mellitus; vs., versus; *, presence versus absent.

**Table 4:** The comparisons of predictive models for HFimpEF.

Predictive Models	Dependent Variable: HFimpEF								
	AUC			Net Reclassification Improvement			Integrated Discrimination Indices		
	M	95% CI	*p* Value	M	95% CI	*p* Value	M	95% CI	*p* Value
Model 1 (NT-proBNP < 1540 pmol/mL)	0.855	0.811–0.892	-	Reference	-		Reference	-	
Model 2 (presence of AF)	0.820	0.715–0.944	0.427	0.10	0.06–0.15	0.388	0.11	0.05–0.17	0.481
Model 3 (LAVI < 39 mL/m^2^)	0.721	0.680–0.773	0.044	0.03	0.01–0.06	0.642	0.06	0.02–0.09	0.552
Model 4 (irisin ≥ 10.8 ng/mL)	0.960	0.910–0.988	0.001	0.36	0.24–0.49	0.001	0.44	0.38–0.52	0.001
Model 1 + model 2	0.848	0.790–0.910	0.066	0.10	0.05–0.17	0.249	0.12	0.06–0.19	0.265
Model 1 + model 3	0.851	0.810–0.912	0.270	0.09	0.03–0.15	0.338	0.11	0.03–0.17	0.286
Model 1 + model 4	0.979	0.932–0.982	0.001	0.38	0.29–0.50	0.001	0.44	0.35–0.54	0.001

Abbreviations: AUC, area under curve; CI, confidence interval; LAVI, left atrial volume index; NT-proBNP, N-terminal brain natriuretic pro-peptide. Note: *p*-value shows a significant difference between variables in terms of Model 1.

## Data Availability

The data presented in this study are available on request from the corresponding author due to privacy restrictions.
